# Role of Receptor Protein Tyrosine Phosphatases (RPTPs) in Insulin Signaling and Secretion

**DOI:** 10.3390/ijms22115812

**Published:** 2021-05-28

**Authors:** Julio Sevillano, María Gracia Sánchez-Alonso, Javier Pizarro-Delgado, María del Pilar Ramos-Álvarez

**Affiliations:** Department of Chemistry and Biochemistry, Facultad de Farmacia, Universidad San Pablo-CEU, CEU Universities, Urbanización Montepríncipe, 28925 Alcorcón, Madrid, Spain; jsevilla@ceu.es (J.S.); msancheza@ceu.es (M.G.S.-A.); javier.pizarrodelgado@ceu.es (J.P.-D.)

**Keywords:** receptor protein tyrosine phosphatases (RPTP), insulin signaling, insulin secretion, T2DM

## Abstract

Changes in lifestyle in developed countries have triggered the prevalence of obesity and type 2 diabetes mellitus (T2DM) in the latest years. Consequently, these metabolic diseases associated to insulin resistance, and the morbidity associated with them, accounts for enormous costs for the health systems. The best way to face this problem is to identify potential therapeutic targets and/or early biomarkers to help in the treatment and in the early detection. In the insulin receptor signaling cascade, the activities of protein tyrosine kinases and phosphatases are coordinated, thus, protein tyrosine kinases amplify the insulin signaling response, whereas phosphatases are required for the regulation of the rate and duration of that response. The focus of this review is to summarize the impact of transmembrane receptor protein tyrosine phosphatase (RPTPs) in the insulin signaling cascade and secretion, and their implication in metabolic diseases such as obesity and T2DM.

## 1. Introduction

### 1.1. Metabolic Diseases Associated with Insulin Resistance and Chronic Low-Grade Inflammation

Type 2 diabetes mellitus (T2DM) and obesity are two major medical challenges of the 21st century with increasing prevalence during the last decades, reaching pandemic proportions [1]. Both metabolic diseases are associated to insulin resistance, and although only a subset of obese people develops T2DM, obesity is a major risk factor for T2DM, and rates of T2DM prevalence have paralleled those of obesity. Consequently, these metabolic diseases, and the morbidity associated with them, accounts for enormous costs for the health systems.

**Figure 1:**
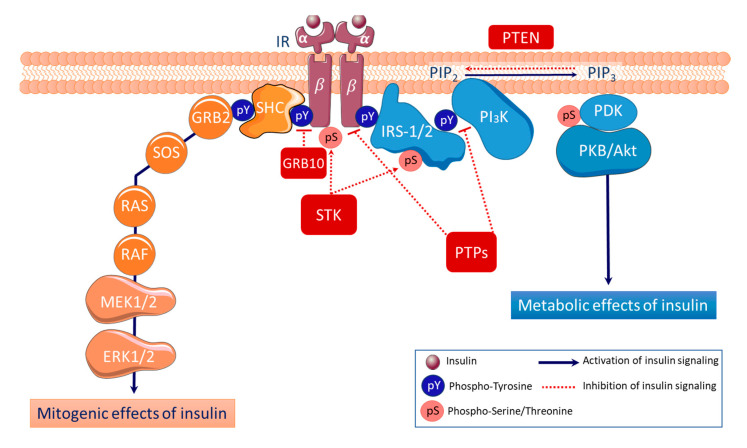
Insulin signaling cascade.

Insulin resistance is a condition in which tissues are unable to respond to normal plasma insulin level, with tissue-specific functional consequences. The integrated physiology of insulin resistance owes to defective insulin action at target cells. Thus, the research on insulin resistance has usually been focused on the critical nodes of the signaling pathway (insulin receptor, insulin receptor substrates (IRS) proteins, protein kinase B (AKT)) described with detail in Figure 1.

Both obesity and T2DM [2] are also associated with the development of a chronic low-grade inflammation in adipose tissue, with local production of proinflammatory cytokines [3], such as tumor necrosis factor-alpha (TNF-*α*) and interleukin-6 (IL-6) [4] that can interfere with insulin receptor signaling (see Figure 1) and cause insulin resistance [5]. Many of these obesity/T2DM-generated inflammatory signals converge to activate serine kinases promoting Ser phosphorylation of IRS1 (pSerIRS1), that directly interferes with insulin action in adipose tissue both in pathological [6] and physiological conditions [7].

Insulin signaling begins with its binding to the cell surface insulin receptor (IR), a heterotetrametric protein (two α extracellular subunits and two transmembrane β-subunits) which is a tyrosine kinase [8]. The binding of insulin to its receptor leads to the activation of IR tyrosine kinase; the receptor is autophosphorylated in key tyrosine (pY) residues of the β-subunits, which function as a docking site for tyrosine phosphorylated adaptor proteins such as insulin receptor substrates 1 and 2 (IRS-1/2) that are subsequently phosphorylated. The recruitment of phosphotyrosine IRS-1/2 to IR leads to the activation of the two major downstream pathways, the phosphatidylinositol-3-kinase (PI_3_K) and the mitogen-activated protein kinase (MAPK) pathway initiated by growth factor receptor bound protein 2 (GRB2) and the Src homology and collagen (SHC). First, activation of the PI_3_K leads to 3-phosphoinositide-dependent protein kinase 1 (PDK1)-dependent phosphorylation and the subsequent activation of the protein kinase B (PKB or AKT), which in turn phosphorylates various key downstream effectors involved in the metabolic effects of the insulin, including glycogen synthase kinase (GSK)-3, Forkhead box protein O (FOXO), and mechanistic target of rapamycin (mTOR). Secondly, activation of the MAPK cascade, which includes rat sarcoma (RAS), rapidly accelerated fibrosarcoma (RAF), mitogen-activated protein/extracellular signal-regulated kinase (MEK1/2), and extracellular signal-regulated kinase (ERK1/2), plays a critical role for mitogenic effects of insulin, including proliferation, differentiation and survival [9]. Insulin signaling is negatively regulated by a number of mechanisms, such as a) the binding of GRB10 to the kinase domain of IR; b) the serine/theonine phosphorylation (pS) of IRS1 by various serine/threonine kinases (STK), including IκB kinase β (IKKβ), c-Jun N-terminal kinase (JNK), S6K and mTOR3 [6]; c) dephosphorylation of IR and IRS1/2 by protein tyrosine phosphatases (PTPs) such as phosphatase-1B (PTP-1B) [10], and d) dephosphorylation of phosphatidylinositol 3,4,5-trisphosphate (PIP_3_) into phosphatidylinositol 4,5-bisphosphate (PIP2) by the phosphatase and tensin homolog (PTEN).

### 1.2. Role of Protein Tyrosine Kinases and Protein Tyrosine Phosphatases Antagonism

Impaired insulin signaling is a common feature of the pathophysiology of human obesity and T2DM. Although, several studies have increased the knowledge regarding the role of reversible tyrosine phosphorylation of the insulin receptor and its substrate proteins in the mechanism of insulin action (Figure 1), it is still necessary to further investigate how the mechanism of insulin action is regulated, or which are molecular defects underlying insulin resistance.

Protein tyrosine kinases (PTKs) catalyze tyrosine phosphorylation, a key reversible post-translational mechanism that is required for metabolic homeostasis, regulation of cell growth and differentiation [11]. This covalent modification is a reversible mechanism of protein regulation in which protein tyrosine kinases catalyze the phosphorylation and protein tyrosine phosphatases (PTPs) are responsible for the removal of the tyrosine-bound phosphate groups [12]. The activities of protein tyrosine kinases and phosphatases are coordinated [13], thus, protein tyrosine kinases amplify the signaling response, whereas phosphatases are required for the regulation of the rate and duration of that response [14].

Aberrant regulation of either PTKs or PTPs has been detected in multiple cancers, often correlating with poor prognosis [15], and changes in tyrosine phosphorylation are associated with cell metabolic disorders. Thus, PTPs have been studied as pharmaceutical targets due to their role in metabolic diseases such as cardiovascular disease, obesity and T2DM [16].

The human genome includes 107 protein tyrosine-phosphatases genes grouped in four different families. The class I of phosphatases includes the 38 classical protein tyrosine-phosphatases and 61 dual-specific tyrosine/serine, threonine phosphatases [17]. Class II is constituted in humans by the low-molecular-weight PTP (LMPTP) [18]. Class III members are the CDC25 cell cycle regulators. In classes I, II and III catalysis is initiated by nucleophilic attack of a cysteine residue on the phosphoryl group of the substrate promoting the subsequent substrate dephosphorylation [19]. Class IV contains the transcription factor Eyes absent (EYA) proteins that have a catalytic mechanism that is not based in the cysteine nucleophilic attack. Based on their overall structure, classical protein tyrosine phosphatases are either non-transmembrane PTPs or transmembrane receptor-like PTPs (RPTPs) [20,21].

The aim of this review is to summarize the impact of the transmembrane receptor-like PTPs (RPTPs) in the insulin signaling cascade and secretion, and their putative implication in metabolic diseases associated to insulin resistance such as obesity and T2DM.

## 2. RPTPs: The Transmembrane Receptor-Like Protein Tyrosine Phosphatases

**Figure 2:**
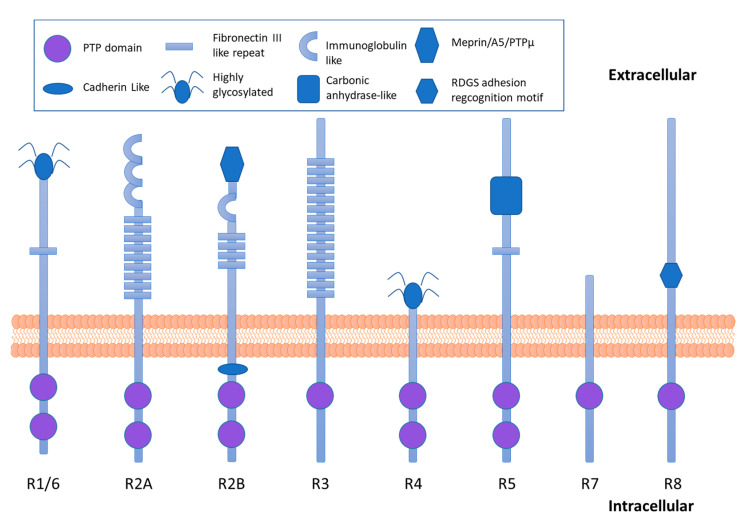
Schematic diagram of the receptor protein tyrosine phosphatases (RPTPs) subfamilies classified to date.

The molecular structure of the RPTPs (Figure 2) allows an interaction with extracellular adhesion molecules and the regulation of intracellular signaling pathways [22]. RPTPs have a variable N-termini extracellular domain with different structural motifs that share homology to cell adhesion molecules, a single transmembrane domain and highly conserved cytoplasmatic domains that in most of the cases contain two PTPs domains (D1 and D2). The protein tyrosine phosphatase domain proximal to the membrane (D1) is responsible for the catalytic activity whereas the protein tyrosine phosphatase domain distal from the membrane has been proposed to have a regulatory function (D2) [14]. Both catalytic and noncatalytic domains participate in the specificity for the substrate, trafficking protein tyrosine phosphatase to subcellular compartments in which concentration of substrate is high, and the catalytic PTP domains dephosphorylate the residues of tyrosine with high selectivity for the substrate [18].

Type R1/6 RPTPs subfamily contains a single fibronectin type III domain (FNIII) extracellularly and two cytoplasmic phosphatase domains. The type R2A RPTPs subfamily has large extracellular domains consisting of three NH_2_-terminal immunoglobulin-like (Ig) domains and nine fibronectin type III domains. Type R2B RPTPs subfamily has an extracellular meprin-A5-PTPµ (MAM) domain, a single Ig domain, and multiple FNIII domains. Type R3 RPTPs subfamily has 15 FNIII domains extracellularly and only one cytoplasmic phosphatase domain. Type R4 RPTPs subfamily have very short extracellular domains, which are often highly glycosylated while type R5 RPTPs subfamily extracellular domains have a carbonic anhydrase domain, linked to a single FNIII domain. Type R7 and R8 RPTPs subfamilies have only one cytoplasmic phosphatase domain. Type R7 RPTPs have a short extracellular domain, whereas type R8 RPTPs contain a peptide Arg-Gly-Asp-Ser (RDGS) adhesion recognition motif.

Several RPTPs have shown to dephosphorylate the insulin receptor and its cellular substrates such as IRS-1, or other tyrosine-phosphorylated proteins along the cellular cascade of insulin action. Since multiple PTPs are expressed in the major insulin-sensitive tissues (i.e., adipose, liver, and muscle), there is a need to ascertain the role of individual RPTPs subfamily in the regulation of insulin signaling (See Table 1).

### 2.1. CD45 and R1/6 Subfamily and Insulin Signaling

The R1/6 subfamily of the RPTP is constituted by CD45, the first RPTP to be identified and that is encoded by the gene *Ptprc* [23]. The extracellular domain is highly glycosylated and contains only one fibronectin type III like domain, whereas two conserved PTP catalytic domains are found in the intracellular region [12] (Figure 2). CD45 is expressed in the surface of nucleated hematopoietic cells and their precursors [24]. In T and B cells CD45 activity is required for antigen receptor-induced tyrosine phosphorylation, phosphatidylinositol (4,5)-bisphosphate hydrolysis, VAV-1 and RAS activation, Ca^+2^ mobilization, development, proliferation and cytokine production [25]. In T and B cells, CD45 modulates tyrosine phosphorylation of SRC kinases. Moreover, CD45 also functions as a Janus kinases (JAKs) phosphatase and negatively regulates cytokine receptor signaling [25]. In accordance, immunoneutralization of CD45 has been shown to inhibit interleukin 4 signal transduction [26].

Other studies have shown that CD45 is a negative modulator of growth factor receptor tyrosine kinases. Transfection of the CD45 into mouse mammary C127 cells decreases PDGF and IGF-1-dependent receptor autophosphorylation and mitogenesis [27]. The expression of CD45 also decreases IGF-1 and PDGF-dependent PI_3_K activation, IRS-1 tyrosine phosphorylation, and their association with signaling complexes [27]. Similar results were observed in 3T3L1 fibroblasts, in the HepG2 hepatocellular carcinoma cell line [28] and in human embryonic kidney fibroblast cells Hek293 [29].

To corroborate if CD45 can also modulate insulin receptor signaling in hematopoietic cells, CD45^−^ and CD45^+^ cells from the insulin-responsive human multiple myeloma cell line (U266 cells) were isolated by fluorescence-activated cell sorting. Insulin receptor autophosphorylation and IRS-1 and SHC phosphorylation were increased in CD45^−^ cells. Moreover, IRS-1/PI_3_K association and MAP kinase activation were increased in CD45 nonexpressing (CD45^-^) cells [28].

### 2.2. LAR and R2A Subfamily and Insulin Signaling

RPTP-δ, RPTP-σ, and Leukocyte common antigen-related (LAR) protein are the members of the R2A subfamily of RPTPs. These RPTPs contain immunoglobulin-like and fibronectin type III like domains as extracellular domains and two PTPs domains in the intracellular fraction (Figure 2).

LAR is a transmembrane PTPase encoded by the *Ptprf* gene and expressed in insulin sensitive tissues like liver, muscle and adipose tissue [30]. Considerable data support the role of LAR as a physiological regulator at a proximal site in the insulin-signaling pathway. Firstly, a physical association between LAR and the insulin receptor has been shown by immunoprecipitation of LAR and immunoblotting with an antibody against insulin receptor, or vice versa. LAR co-immunoprecipitates with insulin receptor [31] and modulates insulin receptor signaling by dephosphorylating regulatory phosphor-tyrosine residues of the insulin receptor [31].

Following auto-phosphorylation of the insulin receptor, the insulin receptor is internalized through an endosomal compartment where it is dephosphorylated prior to being recycled back to the plasma membrane in the basal state. After insulin-stimulation LAR also translocates from the plasma membrane fraction to the endosomal compartment where LAR participates in the dephosphorylation of the insulin receptor [31].

Reduction of LAR protein levels by antisense RNA expression in rat hepatoma cell line increased phosphorylation of the insulin receptor and increased insulin stimulated PI_3_K activity [32]. Moreover, a reduction in the expression of LAR also increased IRS-1 tyrosine phosphorylation, IRS-1 association with PI_3_K and MEK and MAP kinase activities [32].

In humans, insulin resistance and obesity are associated to an increased PTP activity towards the insulin receptor and increased expression of LAR in the subcutaneous adipose tissue. Immunoneutralization of LAR with specific antibodies normalized the PTP activity towards the insulin receptor in the adipose tissue from the obese subjects [33].

On the other hand, transgenic mice that overexpress human LAR in skeletal muscle comparable to those reported in insulin resistant humans exhibit normoglycemia and hyperinsulinemia at the basal state. Moreover, transgenic mice had a reduced whole body glucose disposal and reduced glucose uptake in skeletal muscle compared to wild-type mice. The analysis of insulin signaling revealed that insulin-stimulated phosphorylation of the insulin receptor and IRS-1 in the muscle of transgenic mice was similar to the one of wild-type animals. However, phosphorylation of IRS-2 and IRSs (IRS-I and IRS-2) association with PI_3_K was significantly reduced in muscle of transgenic mice causing whole-body insulin resistance. These observations are positive indicators of insulin resistance and suggest that LAR may be involved in the pathogenesis of T2DM [34].

### 2.3. RPTPκ and R2B Subfamily

The R2B subfamily members of RPTPs display inmunoglobulin, fibronectin type III, and meprin/A5/PTPµ (MAM) domains in their extracellular fraction and two intracellular PTP domains. The R2B subfamily members include RPTP-κ (encoded by the *Ptprk* gene), RPTP-μ (encoded by the *Ptprm* gene), RPTP-π (encoded by the *Ptpru* gene), and RPTP-ρ (encoded by the *Ptprt* gene) [35]. Up to date, no available studies have associated the members of R2B subfamily with insulin action or secretion.

### 2.4. R3 Subfamily and Insulin Signaling

The members of the R3 subfamily of RPTP contain fibronectin type III like domains in the extracellular region, and one phosphatase domain in the intracellular region (Figure 2). The R3 subfamily includes DEP-1 (encoded by the *Ptprj*), PTP-β (encoded by the *Ptprb* gene), SAP1 (encoded by the *Ptprh* gene), mGLEPP (encoded by the *Ptpro* gene in the mouse) and PTPS31 (encoded by the *Ptprp* gene).

In an enzyme–substrate relationships screening, several receptor protein tyrosine kinase (RPTKs) were identified as substrates of the R3 subfamily. These results were further validated by the analysis of in vitro dephosphorylation and overexpression and knock-down experiments in cells [36]. The coexpression of R3 RPTPs with the insulin receptor in HEK 293 cells confirmed that insulin receptor is a substrate for the R3 subfamily members. R3 RPTPs suppress the insulin-induced tyrosine phosphorylation of the insulin receptor by dephosphorylating specific tyrosine residues (Y960 and Y1146). Among the R3 subfamily members, DEP-1 is the only R3 RPTP that is coexpressed with the insulin receptor in the insulin sensitive tissues such as the skeletal muscle, liver and adipose tissue. Moreover, in the liver of *Ptprj*-deficient mice, in which DEP-1 expression is suppressed, glucose and insulin tolerance are increased and insulin-stimulated phosphorylation of insulin receptor and AKT activation are increased [37].

### 2.5. RPTP-α, RPTP-ε and the R4 Subfamily in Insulin Signaling

RPTP-α and RPTP-ε are R4 subfamily members, and they are encoded by the *Ptpra* and *Ptpre* genes, respectively. The members of this subfamily contain a short, highly glycosylated extracellular domain, a membrane-spanning domain, and two cytoplasmic PTP domains (D1 and D2) with structural similarities (Figure 2). As the catalytic activity of D1 is higher than D2, D2 is believed to be involved in substrate recognition and to have a regulatory function [20].

Baby hamster kidney cells (BHK-IR cells) overexpress the insulin receptor and respond to insulin stimulation with complete growth inhibition of adherent cells. RPTP-α and RPTP-ε have shown to act as negative regulators of insulin receptor tyrosine kinase phosphorylation restoring cell growth in BHK-IR cells [38].

*Ptpra* mRNA is widely expressed in mouse tissues with the highest expression in brain and kidney [39]. RPTPα is implicated in the activation of SRC family kinases, and plays a fundamental role in the regulation of integrin signaling [40], cell proliferation and survival [41], and neuronal migration and differentiation [42,43]. Overexpression of RPTPα induces cell transformation and tumorigenesis, exerting at least some of its oncogenic effects through pp60c-SRC kinase [41].

A role for RPTPα in the negative regulation of insulin signaling has also been suggested by studies using overexpression systems [38,44,45]. Thus, in addition to SRC family kinases, some studies have revealed that RPTPα may also dephosphorylate the β subunits of the insulin receptor [44]. In fact, overexpression of RPTPα in rat adipose cells significantly decrease insulin-stimulated recruitment of GLUT4 translocation to the surface of the cells [38]. In contrast, in other studies, RPTPα depletion by antisense mRNA in 3T3-L1 adipocytes did not show any effect in the dephosphorylation of the insulin receptor or the IRSs, in insulin stimulated ERK2 activation or in the insulin-stimulated increase in DNA synthesis [46].

RPTP-ε dephosphorylate insulin receptor in tyrosine residues Y972, Y1158, Y1162 and Y1163 in rat primary hepatocytes. Adenoviral expression of RPTP-ε has shown to decrease tyrosine phosphorylation of insulin receptor and to inhibit insulin-induced activation of its downstream enzymes such as AKT, ERK, and GSK3. As a result, insulin induced glycogen synthesis was completely suppressed in hepatocytes overexpressing RPTP-ε. Moreover, RPTP-ε decreased hepatic glucose output in hepatocytes and decreased in part insulin-induced suppression of the phosphoenolpyruvate carboxykinase (PECK) gene in primary hepatocytes and mouse liver. These results suggest that RPTP-ε is a negative regulator of insulin receptor signaling and participates in the regulation of hepatic glucose metabolism [47].

### 2.6. RPTP- β/ζ, RPTP-γ and the R5 Subfamily in Insulin Signaling

The members of the R5 subfamily include RPTP-β/ζ and RPTP-γ. RPTP-β/ζ is encoded by the *Ptprz* gene [47] and RPTP-γ is encoded by the *Ptprg* gene [48]. These proteins contain an N-terminal carbonic anhydrase like domain, a fibronectin type III like domain, a serine, glycine-rich domain necessary for the chondroitin sulfate attachment, a transmembrane segment and tandem PTP domains (Figure 2).

Although, to our knowledge, there is no study describing a direct interaction of RPTP-β/ζ with insulin receptor signaling, a role for RPTP-β/ζ in regulating IGF-I signaling and cellular proliferation has been proposed [48]. RPTP-β/ζ binds and dephosphorylates PTEN, what increases the dephosphorylation of phosphatidylinositol-3,4,5-triphosphate and inhibits AKT activation (Figure 1). IGFBP-2 can bind, through its unique heparin binding domain, to RPTP-β/ζ and inhibits RPTP- β/ζ what decreases PTEN activity and increases AKT activation inducing cell proliferation [49].

The best-known ligands of RPTP- β/ζ are midkine and pleiotrophin (PTN), which bind to RPTP-β/ζ and inactivate its phosphatase activity. These two cytokines modulate the immune response and/or inflammation in different central disorders characterized by overt neuroinflammation such as neurodegenerative diseases and endotoxemia and are associated with peripheral inflammation and insulin resistance [50]. Regarding the effects on insulin sensitivity, recent studies of our group in a *Ptn* knock-out mice model revealed that deletion of *Ptn* accelerates the development of age related whole-body glucose intolerance, favoring a prediabetic state and predisposing these animals to develop T2DM [51].

Protein tyrosine phosphatase receptor gamma (RPTP-γ) has also shown to be a negative regulator of hepatic insulin signaling in physiological conditions, in obesity and in inflammation in mice. In fact, high fat diet induced obesity or lipopolysaccharide (LPS) treatment have shown to stimulate hepatic RPTP-γ expression in mice. Hepatic RPTP-γ overexpression, to the levels that are observed in obesity, induce the development of hepatic and systemic insulin resistance in mice [52].

In humans, non-alcoholic fatty liver disease (NAFLD) is associated with an increased hepatic expression of RPTP-γ. Moreover, RPTP-γ mRNA levels correlate with the expression of several targets of NF-κB and with the insulin resistance index (HOMA-IR) [52]. These data reflect that RPTP-γ may be a link between obesity and insulin resistance and a new putative target for the treatment of obesity and type 2 diabetes.

### 2.7. R7 Subfamily in Insulin Signaling

The R7 subfamily is constituted by PTPRR and STEP (striatal-enriched protein tyrosine phosphatase). PTPRR (encoded by the *Ptprr* gene) contains a single PTP intracellular domain.

PTPRR have shown to modulate the activity of some members of the mitogen-activated protein kinase (MAPK) pathway. In fact, all PTPRR isoforms contain a kinase interacting motif that catalyzes the dephosphorylation of MAPK. Accumulating evidence supports that tyrosine phosphorylated ERK1/2/5 and p38 are substrates of PTPRR. The interaction of MAPKs to the kinase interacting motif of PTPRR lead to their dephosphorylation and inactivation, and blocks the translocation of the MAP kinases to the nucleus [53].

### 2.8. IA2 and the R8 Subfamily in Insulin Content and Secretion

The human islet antigen-2 (IA2 or ICA512) and IA2β are the transmembrane protein tyrosine phosphatases from the R8 subfamily. IA2 is encoded by the *Ptprn* gene and the *Prprn2* gene encodes IA2β. These RPTPs contain an extracellular RDGS-adhesion recognition motif, a transmembrane domain and an intracellular domain with one PTP domain. This PTP domain has two substitutions in two catalytic residues (Ala911→Asp and Asp877→Ala) that are known to be necessary for the catalytic activity of other PTPs.

The expression of IA2 has been detected in neuroendocrine cells throughout the body and it is an integral component of dense core secretory vesicles of α and β cells of pancreatic islets [43]. IA2 has been extensively studied in human diabetic patients as it is a major autoantigen in type I diabetes, and up to 70% of newly diagnosed type I diabetes patients have autoantibodies to IA2 [54]. As these autoantibodies can be detected years before the onset the clinical disease, antibody screening has been widely used as predictive markers in intervention trials for the prevention of type 1 diabetes [55]. Noteworthy, the expression of IA2 in pancreatic β-cells is induced by glucose, and insulin and decreases by proinflammatory cytokines, whereas IA2β expression is not affected by glucose levels [56].

In IA2-deficient mice, glucose tolerance is impaired, insulin secretion is decreased and in vitro insulin release after glucose stimulation from isolated islets is reduced [57]. Knock-down of endogenous IA2 by short interfering RNA in the glucose-sensitive cloned mouse β-cell line, MIN-6, resulted in a loss of glucose-induced insulin secretion and a 50% decrease in basal insulin release, corroborating the impaired insulin secretion in the IA2-deficient mice [58]. On the other hand, overexpression of IA2 in MIN-6 cells, leads to an increase in glucose-induced and K^+^-induced insulin secretion along with an increase in the number of vesicles, in the content of insulin vesicles in the β-cells and in the half-life of insulin, that is an indicator vesicle stability [58]

As observed in the IA2 deficient mice, IA2β deficient mice also exhibit a mild glucose intolerance and impaired insulin secretion. Glucose-induced insulin secretion in isolated islets from mice IA2β deficient mice was also decreased resembling the results obtained in the IA2 deficient mice, however, a slightly lesser inhibition of glucose-induced insulin secretion was observed in the incubated islets [59]

Furthermore, islets from double knockout (DKO) mice exacerbates the decrease in insulin content, insulin secretion and the half-life and number of dense core secretory vesicles affecting the content and the secretion of insulin in the pancreatic islets from IA2 and IA2β single knock-out mice [60].

## 3. RPTPS as Potential Therapeutic Targets

During several years, the development of potent, selective and cell-permeable useful PTP inhibitors suitable for therapeutic use has proved challenging [61], and highlight that the development of efficient drug delivery systems requires large-scale, multidisciplinary research efforts that combine biological and pharmaceutical biotechnology, and biochemistry sciences.

The RPTP subfamily is unique among PTPs in their potential to be targeted through their intracellular wedge motifs or extracellular regions. Although scarce, recent advances have shown that RPTPS, in particular RPTPβ/ζ (R5 subfamily of RPTP), are potential therapeutic targets for pharmaceutical interventions in central nervous system diseases and as antitumoral therapy. Among them, MY10 is a permeable inhibitor that interacts with the citoplasmatic D1 domain of RPTPβ/ζ and inhibits its tyrosine phosphatase activity [62]. MY10 has shown to reduce alcohol consumption, alcohol-induced conditioned place preference in mice [63] and blocked the alcohol rewarding effects in *Ptn* knockout mice [64]. Another RPTPβ/ζ inhibitor, SCB4380 is a cell-impermeable compound that has shown to suppress the migration and proliferation of glioblastoma cells and tumor growth when delivered intracellularly using cationic liposomes [65]

Compound 211 is a selective, irreversible and noncompetitive inhibitor of CD45-PTP (R1/6 subfamily of RPTP) that promotes antigen receptor signaling in lymphocytes and is considered a drug target for autoimmunity [66]. More recently, pharmacophore modeling, virtual screening and fragment replacement have been successfully applied for the design of a small-LAR (R2a subfamily of RPTP) inhibitor that has shown to strongly interact with the key amino acids of the receptor, and to inhibit enzyme activity [67].

However, to date, there are no studies that have analyzed the potential of RPTPs as therapeutic targets for metabolic diseases, such as diabetes. We believe that this is an area of investigation that deserves attention.

## 4. Concluding Remarks and Future Strategies

This line of evidence supports the role of receptor protein tyrosine phosphatases (RPTPS) in insulin signaling and secretion, and consequently in the pathophysiology of metabolic diseases like T2DM. Accordingly, RPTPS are potential therapeutic targets for pharmaceutical interventions. Therefore, the directed drug design of molecules that mimic ligand binding to the extracellular domains of RPTPs upregulating RPTP activity open new perspectives for the treatment of these metabolic diseases. Given the availability of specific inhibitors, such as MY10, that have been designed through a rational drug program in our group, in ongoing studies we have focused our efforts on this line of research.

## Figures and Tables

**Table 1:** The receptor protein tyrosine phosphatases involved in insulin signaling or secretion.

Family	Representative Phosphatases	Gene	Described Implication in Insulin Action/Secretion
R1/R6	CD45	*Ptprc*	Yes
R2A	LAR	*Ptprf*	Yes
R2B	RPTP-κ	*Ptprk*	No
R3	DEP-1	*Ptprj*	Yes
R4	RPTP-α, RPTP-ε	*Ptpra, Ptpre*	Yes
R5	RPTP-ζ, RPTP-γ	*Ptprz, Ptprg*	Yes
R7	PTPRR	*Ptprr*	Yes
R8	IA2	*Ptprn*	Yes
